# Protective effect of pomegranate seed oil against H_2_O_2_ -induced oxidative stress in cardiomyocytes

**Published:** 2017

**Authors:** Mehdi Bihamta, Azar Hosseini, Ahmad Ghorbani, Mohammad Taher Boroushaki

**Affiliations:** 1 *Department of Pharmacology, Faculty of Medicine, Mashhad University of Medical Sciences, Mashhad, Iran*; 2 *Pharmacological Research Center of Medicinal Plants, Faculty of Medicine, Mashhad University of Medical Sciences, Mashhad, Iran*

**Keywords:** Pomegranate seed, Cardiomyocytes, Reactive oxygen species, Super oxide dismutase, Oxidative stress

## Abstract

**Objective:**

It has been well documented that oxidative stress is involved in the pathogenesis of cardiac diseases. Previous studies have shown that pomegranate seed oil (PSO) has antioxidant properties. This study was designed to investigate probable protective effects of PSO against hydrogen peroxide (H_2_O_2_)-induced damage in H9c2 cardiomyocytes.

**Materials and Methods:**

The cells were pretreated 24 hr with PSO 1 hr before exposure to 200 µM H_2_O_2_. Cell viability was evaluated using 3-(4,5-dimethylthiazol-2-yl)-2,5-diphenyl tetrazolium (MTT) assay. The level of reactive oxygen species (ROS) and lipid peroxidation were measured by fluorimetric methods.

**Results:**

H_2_O_2_ significantly decreased cell viability which was accompanied by an increase in ROS production and lipid peroxidation and a decline in superoxide dismutase activity. Pretreatment with PSO increased viability of cardiomyocytes and decrease the elevated ROS production and lipid peroxidation. Also, PSO was able to restore superoxide dismutase activity.

**Conclusion:**

PSO has protective effect against oxidative stress-induced damage in cardiomyocytes and can be considered as a natural cardioprotective agent to prevent cardiovascular diseases.

## Introduction

Oxidative stress plays a critical role in the pathophysiology of several major cardiovascular diseases such as atherosclerosis, hypertension, heart failure, and myocardial ischemic reperfusion injury (Higashi et al., 2009 ▶). Oxidative stress causes excessive production of reactive oxygen species (ROS), which is an important event in the development of cardiovascular diseases. ROS accumulation may contribute to a number of cardiovascular disorders (Dhalla et al., 2000 ▶). The cellular sources of ROS generation in the heart are including cardiac myocytes, endothelial cells and neutrophils. Within cardiac myocytes, ROS can be derived from mitochondria, xanthine oxidase, NADPH oxidase and uncoupled nitric oxide synthases (Tsutsui et al., 2011 ▶). Increased ROS result in considerable damage to myocardial cells. Antioxidant enzymes such as superoxide dismutase (SOD), catalase (CAT), and glutathione (GSH) play significant role in neutralization of elevated ROS. ROS damage cellular membrane by inducing lipid peroxidation. Malondialdehyde (MDA) is a major lipid peroxidation product and may reflect the degree of cellular injury (Dhalla et al., 2000 ▶). 

Hydrogen peroxide (H_2_O_2_), a major source of ROS can lead to cell membrane injury and cause lipid peroxidation and DNA damage in cells. However, antioxidants can protect cells against H_2_O_2_-induced cell damage via decreasing ROS production (Winstead et al., 2005 ▶; Sun et al., 2012 ▶). Previous studies have shown that some herbal medicine can protect H9C2 cells against H_2_O_2_.

Pomegranate fruit (*Punica granatum* L.) is used as a fruit and has therapeutic effects in traditional medicine. *In vivo* and *in vitro* studies have demonstrated the beneficial effects of pomegranate including anti-microbial, antioxidant, anti-diabetic, and hypolipidemic activities as well as its effect on improving of cardiovascular health (Sadeghian et al., 2011 ▶; Forouzanfar et al., 2013 ▶; Viuda-Martos et al., 2010 ▶). Pomegranate fruit include 78% juice and 22% seed (Kullkarni and Aradhya, 2005 ▶). Pomegranate seeds contain sugars, vitamins, polysaccharides, polyphenols, minerals and low oil (Miguel et al., 2004). Recent studies have found that pomegranate seed is a potential source of nutrients and antioxidants which can be used as a dietary supplement. Recently, studies have shown that pomegranate has several pharmacological activities, such as anti-microbial (El-Sherbini et al., 2010 ▶; Braga et al., 2005 ▶), antioxidant, anti-inflammatory, and anticarcinogenic effects (Lansky and Newman, 2007 ▶). Pomegranate-derived products have shown beneficial effects in the treatment and prevention of cancers, cardiovascular diseases, neurological disorders, diabetes, etc. (Hartman et al., 2006 ▶). Previous investigations have reported that pomegranate seed oil (PSO) causes regeneration of epidermal tissue (Sassano et al., 2009 ▶), boosts the immune system *in vivo*, reduces hepatic triglycerides and has chemopreventive activity against prostate, breast and colon cancers (Caligiani et al., 2010 ▶). Also, it reduces weight gain, and type 2 diabetes risk (Brian et al., 2009 ▶) and improves menopausal symptoms (Lansky and Newmana, 2007 ▶).

The therapeutic effects of pomegranate seeds may be related to the presence of a variety of active compounds, particularly polyphenols, which have antioxidant properties (Balasundram et al., 2006 ▶). Moreover, PSO is a rich source of polyunsaturated fatty acids (PUFAs) including conjugated linolenic acid (CLA) which is an important therapeutic agent that can be used for improvement of human health (Fadavi et al., 2006 ▶)

Based on these findings, we hypothesized that PSO may have protective effect against H_2_O_2_-induced cardiomyopathy. Therefore, the present study was designed to investigate effects of PSO on activity of antioxidant enzyme superoxide dismutase (SOD), lipid peroxidation level and ROS content in H9c2 cardiomyocyte.

## Materials and Methods

**Reagents and chemicals**

H_2_O_2_, 3-(4,5-dimethylthiazol-2-yl)-2,5-diphenyl tetrazolium (MTT), thiobarbituric acid (TBA), 2,7-dichlorofluorescin diacetate (DCFH-DA), High-glucose Dulbecco’s Modified Eagles Medium (DMEM), penicillin-streptomycin and fetal bovine serum were purchased from Gibco. Trichloro acetic acid (TCA) and malondialdehydebis-(dimethyl acetal) (MDA) were obtained from Merck (Darmstadt, Germany). SOD assay kit was purchased from Randox (Autrim, UK). H9C2 cells were obtained from Pasteur Institute (Tehran, Iran). 

**Cell Culture and Treatment**

H9C2 Cells were maintained at 37^o^C in a humidified atmosphere containing 5% CO_2_. The cells were cultured in DMEM supplemented with 10% fetal bovine serum, 100 U/ml penicillin and 100 µg/ml streptomycin. For the experiments, cells were seeded in 96-well and 24-well culture plates for MTT/ROS assay and MDA assay, respectively. After 24 hr, the cells were pretreated (for 24 hr) with PSO (1-100 µM) and then, incubated with 200 µM H_2_O_2_ for 1 hr. Also, cells were exposed to PSO (12-800µg/ml) alone for 24 hr for evaluation of PSO toxicity. 

**Cell viability assay**

Cell viability was determined using a modified 3-(4,5- dimethylthiazol-2-yl)-2,5-diphenyl tetrazolium (MTT) assay as described previously (Mortazavian et al., 2012; Ghorbani et al., 2014). Briefly, MTT solution in phosphate-buffered saline (5 mg/ml) was added to each well at a final concentration of 0.05%. After 3 hr, formazan precipitate was dissolved in DMSO. The absorbance at 570 and 620 nm (background) was measured using a StatFAX303 plate reader. 

**Measurement of reactive oxygen species**

Intracellular ROS levels were evaluated using a fluorescent probe, DCF-DA. At the end of incubation, the cells were treated (30 min) with DCFH-DA (10µM) at 4 ˚C in the dark. Then, the fluorescence intensity was detected at excitation/emission of 485/530 nm. The experiment was performed in triplicate.

**Lipid peroxidation assay**

The level of lipid peroxidation was estimated by measuring MDA which is the end product of lipid peroxidation (Sadeghnia et al., 2013 ▶). At the end of incubation, the cells were scraped and centrifuged for 30 min. Then, 400 µl of TCA (15%) and 800 µl of TBA (0.7%) were added to 500 µl of cell samples. The mixture was vortexed and then, heated for 40 min in a boiling water bath. Then, 200 µl of the sample was transferred to 96-well plate and the fluorescence intensity was read at excitation/emission of 480/530 nm. The experiment was carried out in triplicate.

**Determination of SOD**

The level of SOD activity was determined using a kit which measures SOD activity based on the formation of red formazan from 2-(4-iodophenyl)-3-(4-nitrophe-nol)-5-phenyltetrazolium chloride after reaction with superoxide radicals. At the end of incubation, the cells were harvested with a scraper and centrifuged at 2000 g for 10 min at 4ºC. The supernatant was discarded and the cells were broken using the freeze-thaw method (-20ºC for 20 min, then 37ºC bath for 10 min). Phosphate-buffered saline (1 ml) was added to the cell lysate and the sample was sonicated on an ice bath (60 W with 0.5 sec interval for 15 min). In the final step, cells were centrifuged at 10000 g for 15 min at 4ºC and the optical density of supernatant was read spectrophotometrically at 505 nm. 

**Statistical analyses**

Statistical differences among groups were assessed by one-way ANOVA, followed by the Tukey’s* post hoc* test. The results are shown as mean ± SEM. Values of p less than 0.05 were considered statistically significant.

## Results

**Effect of PSO alone on cell viability**

**Figure 1:**
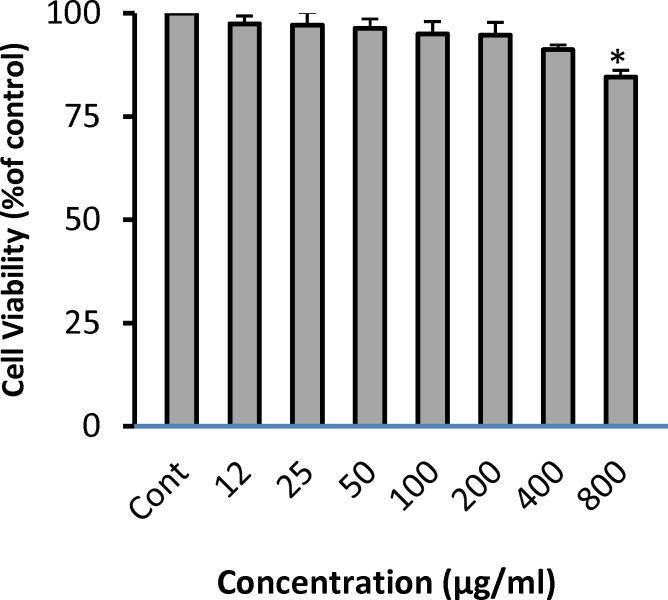
Effect of PSO alone on cell viability in H9c2 cells. The cells were treated (for 24 hr) with different concentrations of PSO. Data are expressed as mean ± SEM of three separate experiments. ^*^p<0.001 vs control

As shown in Figure 1, incubation with PSO for 24 hr significantly decreased the viability of cells at concentration of 800 g/ml (84.5 ± 1.58% of control, p<0.05). Other concentrations did not reduce cell viability. 

**Effect of PSO on cell viability against H**_2_ **O**_2_

**Figure 2:**
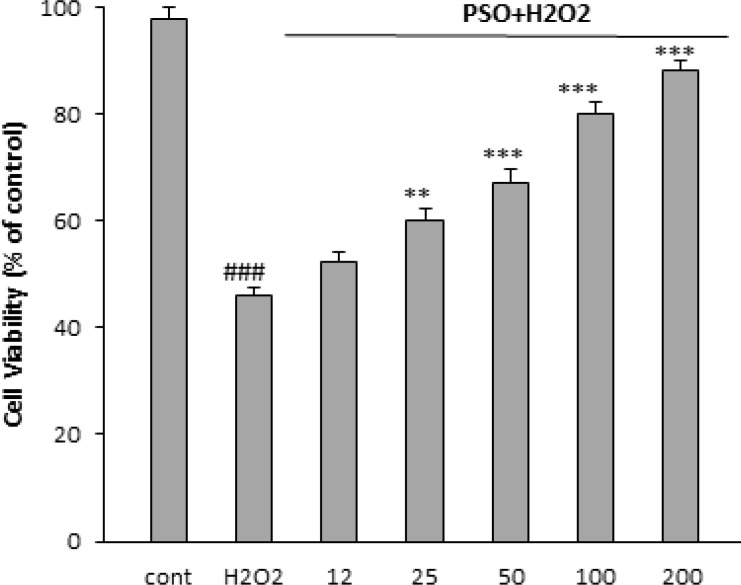
Effect of PSO on H_2_O_2_-induced cytotoxicity in H9c2 cells. The cells were pretreated (for 24 hr) with different concentrations of PSO before to exposure (for 1 hr) to 200 𝜇M of H_2_O_2_. Data are expressed as mean ± SEM of three separate experiments. ### p<0.001 vs control, ** p<0.001 and *** p<0.001 versus H_2_O_2_

Incubation with H_2_O_2_ significantly decreased cell viability to 47 ± 1.5% of control (p<0.001) (Figure 2). Pretreatment with 25, 50, 100 and 200 µg/ml of PSO could increase the viability of H9c2 cells to 60 ± 2.1% (p<0.01), 67± 2.7% (p<0.001), 80.25 ± 2% (p<0.001) and 88± 1.9% (p<0.001), respectively (Figure1). The increase in cell viability at the dose of 12 µg/ml was not significant.

**Effect of PSO on ROS content**

**Figure 3:**
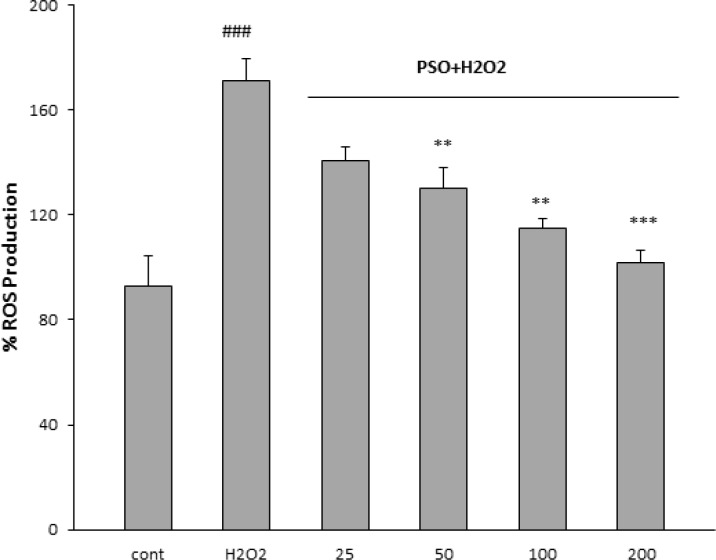
Effect of PSO on H_2_O_2_-induced reactive oxygen species (ROS) generation in H9c2 cells. The cells were pretreated (for 24 hr) with different concentrations of PSO, before exposure (1 hr) to 200 𝜇M of H_2_O_2_. Data are expressed as mean ± SEM of three separate experiments. ### p<0.001 vs control, and **p<0.001 and ***p<0.001 vs H_2_O_2_.

As expected, H_2_O_2_ caused a significant increase in the level of ROS in H9c2 cells as compared to the control (171±8.3%; p<0.001). PSO at concentrations of 50 (130±7.5%, p<0.01); 100 (115±3.6%, p<0.01) and 200 µM (105±4.6%, p<0.001) decreased intracellular ROS level (Figure 3). At concentration of 25 µM did not reduce ROS significantly.

**Effect of PSO on Lipid Peroxidation**

**Figure 4:**
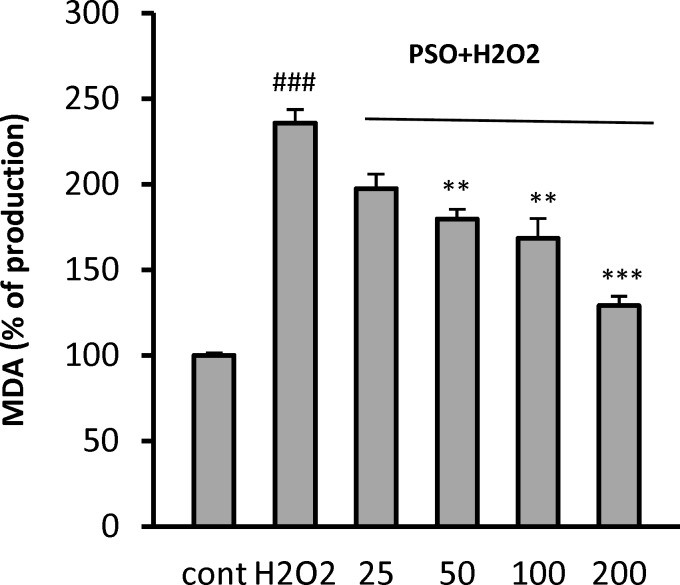
Effect of PSO on H_2_O_2_-induced MDA production in H9c2 cells. The cells were pretreated (for 24 hr) with different concentrations of PSO before exposure (for 1 hr) to 200 𝜇M of H_2_O_2_. Data are expressed as mean ± SEM of three independent experiments. ### p<0.001 vs control, and **p<0.001 and ***p<0.001 vs H_2_O_2_

The level of lipid peroxidation was evaluated by measuring the level of MDA, which is the end product of lipid peroxidation. As shown in Figure 4, exposure of the cells to H_2_O_2_ resulted in a significant increase of MDA level (235.7 ± 7.9%, p<0.001) as compared to control cells (100 ± 1.3%). The content of MDA was significantly decreased in the cells pretreated with 50 (179.8 ± 5.6%, p<0.01), 100 (168.4± 11.7, p<0.01) and 200 µg/ml (129± 5, p<0.001) (Figure4).

**Effect of PSO on Superoxide Dismutase**

**Figure 5:**
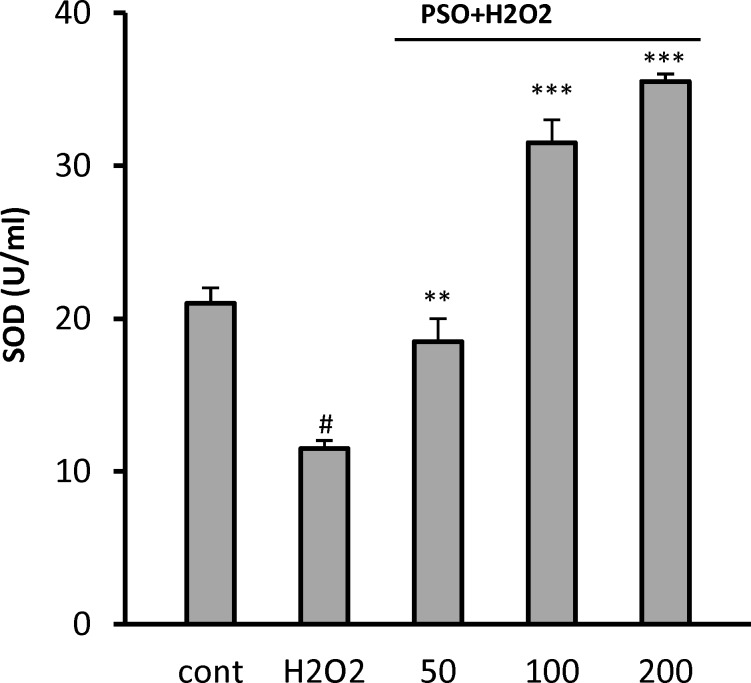
Effect of PSO on superoxide dismutase (SOD) activity following H_2_O_2_ treatment in H9c2 cells. The cells were pretreated (for 24 hr) with different concentrations of PSO before exposure (for 1 hr) to 200 𝜇M of H_2_O_2_. Data are expressed as mean ± SEM of three separate experiments. # p<0.05 vs control, and **p<0.001 and ***p<0.001 vs H_2_O_2_

In order to determine the effect of PSO on cellular antioxidant defenses, the level of SOD was measured (Figure4). H_2_O_2_-induced oxidative stress decreased the level of SOD from 21 ± 1 U/ml (control) to 11.5 ± 0.6 U/ml (p<0.05). As compared to the untreated cells, the level of SOD was significantly increased in pretreated cells at PSO concentrations of 50 (18.5 ± 1.5 U/ml, p<0.01), 100 (31 ± 1.8 U/ml, p<0.001), and 200 µg/ml (35 ± 0.6 U/ml, p<0.001) (Figure 5).

## Discussion

The heart is a target organ for oxidative stress-related injuries. Oxidative stress plays a critical role in the pathophysiology of several major cardiovascular diseases such as atherosclerosis, hypertension, heart failure, and myocardial ischemical reperfusion injury (Higashi et al., 2009 ▶). Poor anti-oxidative capacity of cardiomyocytes subjects them to oxidative damage (Dhalla et al., 2000 ▶). Therefore, a promising approach to cardioprotection is the use of pharmacological means in order to prevent oxidative-damage in the heart. H9c2 is a rat-derived cardiomyoblast cell line that has been used to investigate heart function (L'Ecuyer et al., 2004 ▶). H9c2 cells exhibit morphological characteristics similar to those of immature embryonic cardiomyocytes but preserve several elements of the electrical and hormonal signal pathway found in adult cardiac cells. Evidence have shown that H_2_O_2_ induces the same oxidative stress in H9c2 cells as in primary cultured rat cardiomyocytes (Winstead et al., 2005 ▶). Therefore, this cell line may be useful as a model of oxidative stress-induced cardiomyocyte injury. Antioxidants inhibit or delay oxidation process by preventing the initiation or propagation of oxidizing chain reactions. In the present study, protective effect of PSO on H_2_O_2_-induced oxidative stress was investigated. Our results showed H_2_O_2_ significantly increased the level of ROS, lipid peroxidation and decreased the SOD activity in H9c2 cells. Pretreatment with PSO decreased ROS and MDA production; also, it restored the SOD enzyme activity. Also, treatment of cells with PSO alone did not reduce cell viability except at 800µg/ml. Recent studies have shown that polyphenoles and flavonoids are effective in prevention of some disease such as cardiovascular and inflammatory disorders (Noda et al., 2002 ▶) by inhibition of oxidative stress (Miguel et al., 2004). Previous studies have shown that juice and seed of pomegranate contain antioxidant compounds (Singh et al., 2002 ▶). Studies have shown that antioxidant activity of flavonoids and anthocyanidins in seed oil and juice is three times greater than green tea extract (Okamoto et al., 2004 ▶). PSO has ellagic acid, an antioxidant agent that reduces lipid peroxidation by removing peroxy radical (Ramanathan and Das, 2005 ▶). Moreover, PSO is composed of unsaturated fatty acids as linolenic acids, oleic acid, palmitic acid, and stearic acid. Evidence have suggested that fatty acids with conjugated double bonds may have beneficial effects in inflammation and some types of cancer (Hwang et al., 2007 ▶; Boussetta et al., 2009 ▶) by inhibition of TNFα and prevention of ROS production (Saha and Ghosh, 2009 ▶). Some researchers showed that linolenic acid has antioxidant activity and punicic acid increased antioxidant activity against sodium arsenite-induced oxidative stress (Bouroshaki et al., 2010 ▶; Boroushaki et al., 2013 ▶). Boroushaki and co-workers have shown that PSO has protective effect against hexachlorobutadiene (Boroushaki et al., 2014 ▶) diazinone (Saha et al., 2012 ▶) and mercuric chloride (El-Sherbini et al., 2010 ▶) toxicity in kidney tissue. Tissue protection of PSO may be a result of the presence of antioxidant compounds. Therefore, the protective effect of PSO may be due to the presence of a variety of biologically active compounds. Antioxidant and free radicals scavenging properties of PSO may be partially responsible for its cardioprotective activity. Our results regarding protective action of PSO on cardiomyocyte suggest that it may also induce protective effect against drugs-induced cardiotoxicity. For example, doxorubicin, as an anthracycline antibiotic, is widely used in cancer therapy. However, its clinical usage is limited due to its toxic effect on cardiomyocytes (Xin et al., 2009 ▶). Doxorubicin affects specific enzymes, transporters and metabolic pathways in the cardiac muscle, and may ultimately result in irreversible cardiac failure (Turakhia et al., 2007 ▶). One of the main mechanisms of doxorubicin-induced cardiotoxicity is increase in ROS production (Xin et al., 2009 ▶). According to the results of the present work, PSO antioxidant effect on cardiac cells may reduce cardiotoxic effect of doxorubicin. However, this should be investigated by future studies.

In conclusion, our data demonstrated that PSO has protective effect against hydrogen peroxide-induced cardiomyocyte death. The protective effect was mediated through reducing ROS production and lipid peroxidation. Therefore, PSO may be considered as a natural cardioprotective agent to prevent cardiovascular diseases. But more investigations are needed to elucidate the probable underlying mechanisms of these valuable effects.
